# Childhood family environment and systemic haemodynamics in adulthood: the Cardiovascular Risk in Young Finns Study

**DOI:** 10.1177/14034948241262185

**Published:** 2024-08-17

**Authors:** Erika Kähönen, Terho Lehtimäki, Olli T. Raitakari, Mika Kähönen, Nina Hutri, Liisa Keltikangas-Järvinen, Aino Saarinen

**Affiliations:** 1 Department of Clinical Physiology and Nuclear Medicine, Faculty of Medicine and Health Technology, Tampere University and Tampere University Hospital, Tampere, Finland; 2 Fimlab Laboratories, Tampere, Finland; 3 Department of Clinical Chemistry, Faculty of Medicine and Health Technology, Tampere University and Tampere University Hospital, Tampere, Finland; 4 Finnish Cardiovascular Research Center-Tampere, Tampere University, Tampere, Finland; 5 Centre for Population Health Research, University of Turku and Turku University Hospital, Turku, Finland; 6 Research Centre of Applied and Preventive Cardiovascular Medicine, University of Turku, Turku, Finland; 7 Department of Clinical Physiology and Nuclear Medicine, Turku University Hospital, Turku, Finland; 8 Tampere Centre for Skills Training and Simulation, Faculty of Medicine and Health Technology, Tampere University, Tampere, Finland; 9 Faculty of Medicine, Department of Psychology and Logopedics, University of Helsinki, Helsinki, Finland

**Keywords:** Childhood, socioeconomic family risk, adulthood, haemodynamics

## Abstract

**Aims:**

Childhood family environment is associated with adulthood health behaviours and cardiovascular health, but limited data are available concerning the relationship between childhood family environment and adulthood haemodynamic determinants of blood pressure. We evaluated how childhood family environment predicts adulthood systemic haemodynamics.

**Methods:**

The sample came from the Cardiovascular Risk in Young Finns Study (*n*=1554–1620). Childhood family environment (1980) was assessed with four cumulative risk scores: socioeconomic family risk, risky emotional family atmosphere, stressful life events, and parents’ risky health behaviours. Haemodynamic outcomes in 2007 (participants being 30–45 year-olds) included stroke volume index, systemic vascular resistance index, cardiac output index and heart rate. Analyses were adjusted for childhood (1980) cardiovascular risk factors (high-density lipoprotein and low-density lipoprotein cholesterol, triglycerides, insulin, body mass index and systolic blood pressure); and adulthood (2007) health behaviours (alcohol consumption, smoking, physical activity); and finally for adulthood cardiovascular risk factors.

**Results:**

When adjusted for age and sex, high socioeconomic family risk predicted lower stroke volume index (*P*=0.001), higher heart rate (*P*=0.001) and higher systemic vascular resistance index (*P*=0.030). These associations remained after controlling for childhood cardiovascular covariates or adulthood health behaviours (*P*⩽0.02 for all) but diluted after controlling for adulthood cardiovascular risk factors. The other childhood cumulative risk scores (stressful life events, risky emotional atmosphere, or parents’ risky health behaviour) did not predict adulthood haemodynamic outcomes.

**Conclusions:**

High childhood socioeconomic family risk predicted adulthood haemodynamic outcomes independently of childhood cardiovascular risk factors and adulthood health behaviours, while other childhood psychosocial adversities were not associated with cardiovascular function in adulthood.

## Introduction

Childhood family environment has been found to predict adulthood diabetes [1] and cardiovascular outcomes such as coronary artery calcification [2]. Of the components of childhood family environment, low socioeconomic status [3–5], adverse events [6] and poor parenting practices [7] have been found to predict later cardiovascular outcomes. Considering systemic haemodynamics, lower parental socioeconomic position has been shown to be associated with higher blood pressure from childhood to adulthood [8] and higher heart rate in adulthood [9]. Low childhood socioeconomic status has been reported to predict increased blood pressure by way of negative emotionality and health behaviour [10].

Blood pressure is mainly determined by cardiac output (heart rate × stroke volume) and systemic vascular resistance. High systemic vascular resistance often causes elevated blood pressure [11]. We have recently shown that childhood cardiovascular risk factors such as childhood body mass index (BMI) and lifestyle factors such as physical activity predict systemic vascular resistance in adulthood [12].

To the best of our knowledge, however, very limited evidence has been published concerning the association of childhood family environment and systemic haemodynamic determinants of blood pressure, namely, cardiac output and systemic vascular resistance. There are several potential mechanisms from childhood family environment to systemic haemodynamics later in life. First, childhood family environment may lead to: (a) alterations in childhood cardiovascular risk factors such as blood pressure; (b) certain (un)favourable health behaviours in adulthood such as lack of physical activity, smoking, or consumption of alcoholic beverages; or (c) changes in adulthood cardiovascular factors. Any of these alterations, in turn, may modify systemic haemodynamics later in life.

In the current study, we aimed to investigate the association of childhood family environment with adulthood systemic haemodynamics, taking simultaneously into consideration the afore-mentioned possible mediating factors. For this purpose, we investigated the effects of childhood family environment on cardiac output and systemic vascular resistance in the prospective Cardiovascular Risk in Young Finns Study. Although the study cohort has a 27-year follow-up from childhood to adulthood, the incidence of cardiovascular events is still quite low due to the young age of the cohort. Therefore, we focused on systemic haemodynamics in adulthood in the current study. Childhood family environment was assessed through cumulative scores of socioeconomic family risk, disadvantageous emotional family atmosphere, stress-prone life events, and parents’ risky health behaviours.

## Methods

### Participants

The sample came from the Cardiovascular Risk in Young Finns Study (YFS) that is an on-going follow-up study. The baseline measurement was conducted in 1980 (*n*=3596, composed of six age cohorts born in 1962–1977), and follow-up studies have been conducted since then. The sampling was designed to include a population-based sample of non-institutionalised Finnish children, representative with regard to sex (male vs. female), rural versus urban environment, and eastern versus western regions in Finland. The design of the YFS is described with further details elsewhere [13].

The study design was approved by the ethical committees of all Finnish universities with a medical faculty (Universities of Helsinki, Turku, Tampere, Kuopio and Oulu). All the participants or their parents (if participants were aged <18 years) provided informed consent before participation. The Declaration of Helsinki has been followed throughout the study.

In the analyses of the present study, we included all the YFS participants who had data available on the study variables: childhood family environment in 1980/1983 (participants were 3–18 year-olds), haemodynamic variables in 2007 (participants were 30–45 year-olds), cardiovascular covariates in childhood (1980) and adulthood (2007), and health behaviour covariates in adulthood (2007). Thus, the final sample size was 1554–1620 participants in the analyses (with different blocks of covariates).

### Measures

#### Childhood family environment

Childhood family environment was assessed with four cumulative scores: (a) socioeconomic family risk; (b) disadvantageous emotional family atmosphere; (c) stress-prone life events; and (d) parents’ risky health behaviours.

All the questionnaires related to childhood family environment were completed by a parent (mostly mother) in 1980. In case there were missing values in 1980, we imputed them using data from the closest possible follow-up point (in 1983). We prioritised the year 1980 variables because we wanted to measure family environment as early as possible. In case a participant had missing data both in 1980 and 1983, the participant was dropped out from the analyses.

The cumulative risk score of socioeconomic family risk included the following factors: parents’ occupational status (1 = upper-grade non-manual worker; 2 = lower-grade non-manual worker; 3 = manual worker manual worker); parents’ educational level (1 = academic level; 2 = high school or occupational school; 3 = comprehensive school; i.e. the first 9 years of school); family income before tax (1 = >100 000 Finnish marks, 4 = 45 001–55 000 Finnish marks, 8 = <15 000 Finnish marks; one euro corresponded to 5.94 Finnish marks when Finland changed its currency in 1999); unstable employment situation (1 = at least one parent was unemployed or on a long-term sick leave; 0 = other employment situations); and over-crowded apartment (family size in relation to number of rooms at home). Each item was standardised by age cohort (mean (M) = 0, standard deviation (SD) = 1 within each age cohort), and we calculated a mean score of the standardised items.

The cumulative risk score of adverse emotional family atmosphere included the following factors: emotional distance between the child and parent; parental intolerance towards the child; strict discipline towards the child; parental life dissatisfaction; mother’s or father’s self-reported mental disorder (no/yes); and mother’s or father’s self-reported frequent alcohol intoxication (1 = never; 8 = daily). Parental life dissatisfaction was assessed with a questionnaire adapted from the operation family study questionnaire [14]. Emotional distance between the child and parent, parental intolerance towards the child, and strict discipline towards the child were assessed with scales that are described more thoroughly elsewhere [15–17]. Each factor was standardised by age cohort (M = 0, SD = 1 within each age cohort), and we calculated a mean score of the standardised items.

The cumulative risk score of stressful life events included the following factors: number of changes of residence (a continuous response); number of changes of school (a continuous response); parental divorce (whether parents living together or had separated); mother’s or father’s death (yes/no); mother’s or father’s hospitalisation within the past 12 months (number of days in hospital, ranging from ‘1 = no days’ to ‘5 = more than 30 days’); and child’s hospitalisation due to sickness or accident (yes/no). Each item was first standardised by age cohort (M = 0, SD = 1 within each age cohort) and, then, we calculated a mean score of the standardised items.

The cumulative risk score of parents’ risky health behaviours included the following factors: mother’s and father’s smoking (1 = never, 2 = every now and then, 3 = daily); alcohol consumption (1 = never, 8 = daily); regular physical activity (1 = no regular physical activity, 8 = daily); and BMI. Each type of health behaviour was calculated separately for both the mother and father. Each item was standardised by age cohort (M = 0, SD = 1 within each age cohort), and we calculated a mean score of the standardised items.

Thus, our cumulative risk scores include a range of strengths: (a) a maximally broad scope of family environmental factors; (b) a large sample size (we combined data from the measurements of 1980 and 1983); (c) a utilisation of the original scales of the variables (no cut-off points, no dichotomisations); (d) the factors were standardised within age cohorts in order to control for possible cohort-specific differences. The same scores have also been used previously [15, 18, 19].

#### Haemodynamic outcomes

Haemodynamic outcome variables included stroke volume index (SI), systemic vascular resistance index (SVRI), cardiac output index (CI) and heart rate (HR). Participants were instructed to avoid heavy exercise and alcohol on the previous evening and smoking, caffeine-containing products and heavy meals on the investigation day. Participants lay in the supine position for at least 15 min before the measurement, during which period electrodes for whole-body impedance cardiography were placed on the body surface. A whole-body impedance cardiography device (CircMon B202, JR Medical Ltd., Tallinn, Estonia) was used to determine beat-to-beat HR, SI (stroke volume/body surface area, ml/m^2^), CI (cardiac output/body surface area, l/min/m^2^) and SVRI (systemic vascular resistance/body surface area, dyn·s/cm^5^/m^2^). Briefly, CircMon records the continuous changes in body electrical impedance during a cardiac cycle. The stroke volume and cardiac output values measured with CircMon agree well with the values measured by the thermodilution method and three-dimensional echocardiography [20–22], and the repeatability of measurements by the impedance method has been reported to be better than by the thermodilution method [20]. For further details, please see previous publications [20, 21].

#### Childhood and adulthood cardiovascular risk factors and adulthood health behaviours as covariates

Standard methods were used to determine childhood and adulthood blood pressure, high-density lipoprotein (HDL) cholesterol, low-density lipoprotein (LDL) cholesterol, triglycerides and insulin after an overnight fast, as previously described in detail [23]. BMI was calculated as weight in kilograms divided by height in meters squared. Adulthood health behaviours included physical activity, alcohol consumption and smoking status. Physical activity was evaluated with self-reported frequency and intensity of physical activity, frequency of vigorous physical activity, hours spent on vigorous physical activity, average duration of a physical activity session, and participation in organised physical activity. Alcohol consumption was assessed as self-reported consumption of ^1^/_3_ litre cans or bottles of beer, glasses (12 cl) of wine, and 4 cl shots of liquor or strong alcohol (these portions are comparable to *c*. 14 g of alcohol) within the past week. A sum score of the consumption of different beverages was calculated. Smoking status was dichotomised (1 = daily smoking, 0 = not daily smoking) as has also been done in previous YFS studies [15]. For further details, please see previous publications [24].

### Statistical analyses

We used linear regression analyses to investigate the associations of childhood family environment (1980) with haemodynamic outcomes in adulthood (2007). We predicted each haemodynamic variable separately (SI, SVRI, CI and HR). The childhood cumulative risk scores were added as predictors simultaneously (socioeconomic family risk, adverse emotional family atmosphere, stressful life events, parents’ risky health behaviours). Age and sex were controlled for in all the analyses. In addition, we added other covariates as separate blocks to the analyses: block 1 included only age and sex; block 2 included childhood cardiovascular covariates (HDL and LDL cholesterol, triglycerides, insulin, BMI and systolic blood pressure in 1980); block 3 included adulthood health behaviours (alcohol consumption, daily smoking status, physical activity in 2007); and block 4 included adulthood cardiovascular covariates (HDL and LDL cholesterol, triglycerides, insulin, BMI and systolic blood pressure in 2007). In order to determine whether adulthood BMI and systolic blood pressure mediated the associations between childhood family environment and adulthood systolic haemodynamics, block 4 was analysed also including only adulthood HDL and LDL cholesterol, triglycerides and insulin as covariates, and including adulthood HDL and LDL cholesterol, triglycerides, insulin and BMI as covariates, and finally including adulthood HDL and LDL cholesterol, triglycerides, insulin, BMI and systolic blood pressure as covariates.

In addition, we examined attrition between included and dropped out participants (who were not included due to missing values in the study variables or non-participation in the follow-up study in 2007). This was done using chi square tests (categorical variables) or independent samples t-tests (continuous variables).

The data were analysed using STATA MP version 17.0.

## Results

Descriptive statistics of the study sample (*n* = 1620) are presented in Table I. First, we examined whether participant attrition over the follow-up measurements resulted in biases in the study variables. The results can be found in Supplemental Table I. To summarise, when compared with dropped out participants, included participants were more likely to be women (55.8% vs. 47.0%) and had a slightly more favourable childhood family environment (in terms of lower socioeconomic family risk, less adverse emotional family atmosphere, and fewer stressful life events). In addition, included participants had slightly better cardiovascular health in adulthood: lower SVRI (2685.8 vs. 2782.4), lower systolic blood pressure (120.2 vs. 122.4), lower HDL cholesterol (1.3 vs. 1.4), lower LDL cholesterol (3.1 vs. 3.2) and a lower level of triglycerides (1.35 vs. 1.53) than dropped out participants. We did not find any attrition bias in SI, HR, CI or in most of the childhood cardiovascular covariates, or in adulthood health behaviours.

**Table I.** Childhood family environment and systemic haemodynamics in adulthood: the Cardiovascular Risk in Young Finns Study, descriptive statistics of the study variables.

	Mean (SD)	Frequency (%)	Range (min; max)
Age (years)	37.3 (5.0)		30; 45
Sex (female)		904 (55.8)	
Haemodynamics in adulthood (2007)			
Stroke volume index (ml/m^2^)	42.6 (6.0)		25; 66
Systemic vascular resistance index (dyn·s/cm^5^/m^2^)	2685.8 (538.0)		1327; 4984
Heart rate (beats/min)	65.6 (9.7)		40; 106
Cardiac output index (l/min/m^2^)	2.8 (0.5)		1; 48
Cumulative risk scores of family environment (1980)			
Socioeconomic family risk	–0.1 (0.6)		–1.3; 2.9
Adverse emotional family atmosphere	–0.1 (0.4)		–1.0; 2.7
Stressful life events	–0.1 (0.3)		–0.5; 2.0
Parents’ risky health behaviours	0.0 (0.4)		–1.1; 1.5
Cardiovascular health in childhood (1980)			
Systolic blood pressure (mmHg)	111.9 (11.8)		68; 174
HDL cholesterol (mmol/l)	1.5 (0.3)		0.7; 2.6
LDL cholesterol (mmol/l)	3.3 (0.7)		1.1; 6.2
Triglycerides (mmol/l)	0.6 (0.3)		0.1; 2.6
Insulin (mU/l)	9.6 (6.1)		0; 40
BMI (kg/m^2^)	17.8 (3.0)		9.2; 36.9
Cardiovascular health in adulthood (2007)			
Systolic blood pressure (mmHg)	120.2 (14.2)		77; 199
HDL cholesterol (mmol/l)	1.3 (0.3)		0.6; 3.5
LDL cholesterol (mmol/l)	3.1 (0.8)		0.5; 6.6
Triglycerides (mmol/l)	1.4 (0.8)		0.3; 8.7
Insulin (mU/l)	9.5 (18.4)		0; 654
BMI (kg/m^2^)	25.9 (4.6)		16.6; 53.1
Health behaviours in adulthood (2007)			
Alcohol use (units/week)	0.9 (1.5)		0; 28.6
Physical activity index (points)	8.8 (1.8)		5; 15
Daily smoking (yes)		304 (18.9)	

*n*=1620.

BMI: body mass index; HDL: high-density lipoprotein; LDL: low-density lipoprotein; SD: standard deviation.

In this table, we included all the participants who were included in at least one analysis.

**Figure 1.**
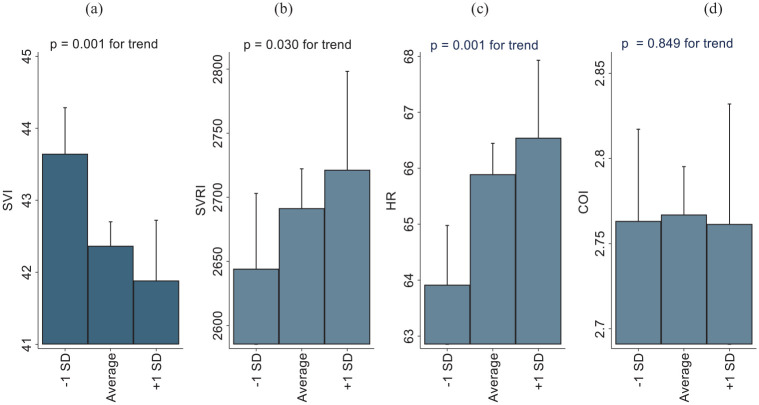
Childhood family environment and systemic haemodynamics in adulthood: the Cardiovascular Risk in Young Finns Study. Estimated means of (a) stroke volume index (SI); (b) systemic vascular resistance index (SVRI); (c) heart rate (HR); and (d) cardiac output index (CI) at low (–1 standard deviation (SD)), average, and high (+1 SD) levels of family socioeconomic risk.

Estimated means of SI, SVRI, HR and CI at low (-1 standard deviation (SD)), average, and high (+1 SD) levels of socioeconomic risk are shown in Figure 1. The results of linear regression analyses are presented in Table II. When adjusted for age and sex (model 1), socioeconomic family risk predicted lower SI (*P*=0.001), higher HR (*P*=0.001) and higher SVRI (*P*=0.030). These associations survived after controlling for childhood cardiovascular covariates in model 2 (*P*=0.001 for SI, *P*=0.002 for HR and *P*=0.038 for SVRI) and after controlling for adulthood health behaviours in model 3 (*P*=0.001, *P*=0.001 and *P*=0.022 for SI, HR and SVRI, respectively). However, all these associations diluted to non-significant after adjusting for adulthood cardiovascular covariates in model 4 (*P*=0.08, *P*=0.09 and *P*=0.78 for SI, HR and SVRI, respectively) (Table II).

**Table II.** Childhood family environment and systemic haemodynamics in adulthood: the Cardiovascular Risk in Young Finns Study, results of regression analyses when predicting haemodynamic outcomes in 2007 by cumulative risk scores of family environment in 1980.

	Model 1 (*n*=1620)			Model 2 (*n*=1580)			Model 3 (*n*=1554)			Model 4 (*n*=1573)		
	B	SE	*P* value	B	SE	*P* value	B	SE	*P* value	B	SE	*P* value
Stroke volume index (SI)												
Socioeconomic family risk	–0.78	0.23	0.001	–0.79	0.20	0.001	–0.76	0.23	0.001	–0.39	0.23	0.080
Stressful life events	–0.42	0.43	0.329	–0.41	0.44	0.351	–0.34	0.44	0.442	–0.05	0.42	0.914
Risky emotional atmosphere	0.56	0.35	0.105	0.57	0.35	0.105	0.55	0.36	0.124	0.42	0.34	0.219
Parents’ risky health behaviour	–0.08	0.40	0.836	–0.07	0.40	0.856	–0.34	0.41	0.409	0.31	0.39	0.427
Systemic vascular resistance index (SVRI)												
Socioeconomic family risk	45.43	20.92	0.030	43.89	21.17	0.038	49.39	21.55	0.022	–5.65	20.09	0.779
Stressful life events	–21.81	39.18	0.578	27.02	40.06	0.500	–24.78	40.09	0.537	–34.05	37.42	0.363
Risky emotional atmosphere	–48.23	31.93	0.130	–36.84	32.26	0.254	–54.64	32.82	0.096	–26.56	30.16	0.379
Parents’ risky health behaviour	33.08	36.36	0.363	23.47	37.12	0.527	46.49	37.55	0.216	17.17	34.67	0.621
Heart rate (HR)												
Socioeconomic family risk	1.27	0.38	0.001	1.18	0.38	0.002	1.25	0.38	0.001	0.64	0.37	0.087
Stressful life events	0.94	0.71	0.184	0.91	0.72	0.207	0.96	0.72	0.181	0.39	0.69	0.575
Risky emotional atmosphere	–0.15	0.57	0.801	–0.07	0.58	0.901	–0.24	0.59	0.679	–0.00	0.56	0.998
Parents’ risky health behaviour	–0.34	0.66	0.604	–0.21	0.67	0.756	–0.16	0.67	0.811	–0.61	0.64	0.344
Cardiac output index (CI)												
Socioeconomic risk	0.00	0.02	0.840	0.00	0.02	0.980	0.00	0.02	0.840	0.00	0.02	0.857
Stressful life events	0.01	0.04	0.851	0.01	0.04	0.868	0.01	0.04	0.744	0.01	0.04	0.830
Risky emotional atmosphere	0.04	0.03	0.207	0.04	0.03	0.177	0.03	0.03	0.280	0.03	0.03	0.262
Parents’ risky health behaviour	–0.02	0.03	0.463	–0.02	0.03	0.544	–0.03	0.03	0.315	–0.01	0.03	0.747

Model 1: Adjusted for age and sex.

Model 2: Adjusted for age, sex and childhood cardiovascular covariates (systolic blood pressure, high-density lipoprotein (HDL) and low-density lipoprotein (LDL) cholesterol, triglycerides, insulin and body mass index (BMI)).

Model 3: Adjusted for age, sex and adulthood health behaviours (alcohol consumption, daily smoking status, physical activity).

Model 4: Adjusted for age, sex and adulthood cardiovascular covariates (systolic blood pressure, HDL and LDL cholesterol, triglycerides, insulin and BMI).

We performed additional analyses to determine whether adulthood BMI and systolic blood pressure partially mediated the associations between childhood family environment and adulthood systolic haemodynamics. When block 4 was analysed including only adulthood HDL and LDL cholesterol, triglycerides and insulin as covariates, socioeconomic family risk predicted lower SI (*P*=0.006), higher HR (*P*=0.004) but not SVRI (*P*=0.082) (Supplemental Table II). When block 4 was analysed including adulthood HDL and LDL cholesterol, triglycerides, insulin and BMI as covariates, socioeconomic family risk still predicted lower SI (*P*=0.04), higher HR (*P*=0.014) but not SVRI (*P*=0.378) (Supplemental Table III). Adjustment for adulthood BMI reduced the regression coefficient between socioeconomic family risk and SI by 26% (from B=–0.621 to B=–0.46) and between socioeconomic family risk and HR by 14% (from B=1.086 to B=0.93). Further adjustment of model 4 for adulthood systolic blood pressure reduced the regression coefficient between socioeconomic family risk and SI by 15% (from B=–0.46 to B=–0.39) and between socioeconomic family risk and HR by 31% (from B=0.93 to B=0.64).

Socioeconomic family risk did not predict CI in adulthood with any block of control variables (*P*=0.177–0.980 in models 1–4).

The other childhood cumulative risk scores, that is, stressful life events, risky emotional atmosphere, or parents’ risky health behaviour did not predict any of the haemodynamic outcomes (*P*=0.096–0.998 in models 1–4).

As additional analyses, we re-ran the main analyses so that each childhood cumulative risk score was included as a predictor separately. The results are shown in Supplemental Table IV. To summarise, all the results were replicated: socioeconomic family risk predicted SI, SVRI and HR but not CI. The other childhood cumulative risk scores did not predict any of the haemodynamic outcomes.

As second additional analyses, we re-ran full-adjusted analyses: that is, we adjusted the associations for all the blocks simultaneously. The results can be found in Supplemental Table V. Briefly, socioeconomic family risk predicted SI and HR but not SVRI or CI. Any other childhood cumulative risk score did not predict the haemodynamic outcomes.

As final additional analyses, we examined possible sex differences in the associations between childhood cumulative risk scores and haemodynamic outcomes. That is, we added sex interaction separately with each childhood cumulative risk score as a predictor in the model. All the sex interactions were non-significant (*P*=0.086–0.983). Thus, the association seemed to be similar in men and women and we did not run the analyses separately in both sexes.

## Discussion

The current study showed that high socioeconomic family risk predicted adulthood lower SI, higher HR and higher SVRI. The associations with SI and HR remained after controlling for childhood cardiovascular risk factors and adulthood health behaviour, but diluted after adjusting for adulthood cardiovascular risk factors. However, socioeconomic family risk did not predict CI in adulthood. The other childhood cumulative risk scores (stressful life events, risky emotional atmosphere, or parents’ risky health behaviour) did not predict adulthood haemodynamic outcomes.

To the best of our knowledge, no evidence has been published concerning the association of childhood family environment and main determinants of adulthood blood pressure (systemic vascular resistance and cardiac output). Some Nordic studies have addressed similar questions using different outcomes. For example, social adversity in adolescence (in women) and young adulthood (in men) was related to allostatic load, independently of cumulative socioeconomic disadvantage and later adversities in adulthood [25]. The present study adds to the current knowledge by showing that high childhood socioeconomic family risk is associated with adulthood small artery status (systemic vascular resistance) and components of cardiac output (stoke volume and HR) but not with cardiac output itself.

Increased resting HR is known to predict several cardiovascular outcomes such as myocardial infarction, stroke, mortality and sudden cardiac death [26, 27]. A previous study reported that disadvantaged childhood socioeconomic position predicts higher adulthood HR independently of smoking status [9]. Interestingly, the current study extended this finding by showing that high socioeconomic family risk predicted adulthood higher resting HR independently of several adulthood health behaviour factors known to have influence on heart rate (physical activity, alcohol consumption, and smoking status).

In the current study, high socioeconomic family risk also predicted lower adulthood SI independently of several childhood and adulthood cardiovascular risk factors and adulthood health behaviour factors. Although the mechanism behind this association is not clear, high socioeconomic family risk could produce cardiac structural and functional alterations leading to diminished SI, or lower SI could just be caused by increased arterial load due to increased systemic vascular resistance. The former mechanism seems more likely as a previous report from the same study cohort showed that low family socioeconomic status (based on annual income) predicts increased left ventricular mass and impaired diastolic performance more than three decades later [28].

As high childhood socioeconomic family risk predicted lower SI and higher HR in adulthood, we consistently did not find any significant effect on adulthood CI (calculated as SI × HR). As socioeconomic family risk predicted SVRI but not CI in adulthood, the present findings suggest that socioeconomic family risk may have a more consistent impact on blood pressure regulation through influences on vascular resistance than on cardiac output.

A previous study from the same cohort showed that early socioeconomic disadvantage influences later blood pressure, in part through blood pressure in early life (that tracks into adulthood) and in part through adulthood BMI [8]. Interestingly, the present study showed that childhood socioeconomic family risk predicted adulthood haemodynamic outcomes also after controlling for childhood cardiovascular risk factors (such as blood pressure) and adulthood health behaviours, suggesting that these factors did not mediate the association between childhood socioeconomic risk and adulthood haemodynamics. However, the findings indicated that the associations between childhood socioeconomic risk and adulthood haemodynamics were at least partially mediated by adulthood cardiovascular risk factors. That is, the association of childhood socioeconomic risk with SVRI diluted to non-significant already after adjustment with adulthood HDL and LDL cholesterol, triglycerides and insulin, while the associations with SI and HR still remained significant. A further adjustment for adulthood BMI reduced the regression coefficient between socioeconomic family risk and SI by 26%, and between socioeconomic family risk and HR by 14%, but the association between socioeconomic family risk and the haemodynamic variables remained significant. A further adjustment for adulthood systolic blood pressure reduced the regression coefficient between socioeconomic family risk and SI by 15% and between socioeconomic family risk and HR by 31%, and diluted these associations to non-significant. Thus, adulthood cardiovascular risk factors, especially BMI and systolic blood pressure, appeared at least partially to mediate the association between socioeconomic family risk and these haemodynamic variables.

Interestingly, the other childhood cumulative risk scores such as stressful life events or risky emotional atmosphere did not predict adulthood haemodynamic outcomes. This is in accordance with previous studies using the same cohort, showing that childhood emotional atmosphere does not predict cardiovascular health index in adulthood [29], or carotid intima-media thickness (a structural marker of early atherosclerosis) in adulthood [30]. On the other hand, adverse childhood family environment in terms of, for example, maltreatment or mother’s expressiveness have been linked to haemodynamic responses to stress in children [31, 32]. Therefore, the present findings provide hopeful evidence that childhood psychosocial adversities may not have long-lasting or stable influences on adulthood systemic haemodynamics. On the other hand, when life events were analysed in a long-term perspective, a cohort study showed that 18 and 30-year-old men with high blood pressure reported few positive life events during their lives or life events in general within the past year [33, 34]. Also, of note, the aim of the current study was to evaluate the associations of childhood ordinary life (i.e. not traumatic level) psychosocial environment with adulthood haemodynamics in a large population cohort. More severe types of childhood stress such as abuse may have longer-lasting influences on adulthood haemodynamics. Altogether, the present findings significantly expand current knowledge regarding the influences of childhood psychosocial risk on cardiovascular health, especially on systemic haemodynamics.

Concerning study limitations, non-invasive haemodynamic measurement findings should always be interpreted with caution. The current method, however, is carefully validated [25–27] and, therefore, well suited for the evaluation of systemic haemodynamics in an epidemiological study setting. Second, our study cohort was ethnically homogenous, consisting solely of white European subjects. As ethnic differences in the haemodynamic mechanisms of blood pressure regulation have been reported [35], the results may not be generalisable to other ethnicities. The strength of our study is the large cohort of young adults followed for 27 years since childhood. Other strengths of the current study include a comprehensive evaluation of the childhood psychosocial environment and a detailed collection potentially confounding lifestyle and cardiovascular risk factors.

The present study has demonstrated that high childhood socioeconomic family risk predicted several haemodynamic outcomes, such as heart rate and systemic vascular resistance in adulthood, independently of childhood cardiovascular risk factors and adulthood health behaviours. The association was diluted after further controlling with adulthood cardiovascular risk factors, suggesting that the association was at least partially mediated through influences on adulthood cardiovascular risk factors. Interestingly, childhood stressful life events, risky emotional atmosphere, or parents’ risky health behaviour did not predict adulthood haemodynamic outcomes.

## Supplemental Material

sj-docx-1-sjp-10.1177_14034948241262185 – Supplemental material for Childhood family environment and systemic haemodynamics in adulthood: the Cardiovascular Risk in Young Finns Study Supplemental material, sj-docx-1-sjp-10.1177_14034948241262185 for Childhood family environment and systemic haemodynamics in adulthood: the Cardiovascular Risk in Young Finns Study by Erika KÄhÖnen, Terho LehtimÄki, Olli T. Raitakari, Mika KÄhÖnen, Nina Hutri, Liisa Keltikangas-JÄrvinen and Aino Saarinen in Scandinavian Journal of Public Health

## References

[1] Pulkki-Råback L Elovainio M Hakulinen C , et al. Positive psychosocial factors in childhood predicting lower risk for adult type 2 diabetes: the Cardiovascular Risk in Young Finns Study, 1980–2012. Am J Prev Med 2017;52:e157–e164. 20170309. DOI: 10.1016/j.amepre.2017.01.042 28284747

[2] Juonala M Pulkki-Råback L Elovainio M , et al. Childhood psychosocial factors and coronary artery calcification in adulthood: the Cardiovascular Risk in Young Finns Study. JAMA Pediatr 2016;170:466–472. DOI: 10.1001/jamapediatrics.2015.4121 26974359

[3] Kivimäki M Smith GD Juonala M , et al. Socioeconomic position in childhood and adult cardiovascular risk factors, vascular structure, and function: Cardiovascular Risk in Young Finns Study. Heart 2006;92:474–480. 20050913. DOI: 10.1136/hrt.2005.067108 16159979 PMC1860895

[4] Poulton R Caspi A Milne BJ , et al. Association between children’s experience of socioeconomic disadvantage and adult health: a life-course study. Lancet 2002;360:1640–1645. DOI: 10.1016/s0140-6736(02)11602-3 12457787 PMC3752775

[5] Clark AM DesMeules M Luo W , et al. Socioeconomic status and cardiovascular disease: risks and implications for care. Nat Rev Cardiol 2009;6:712–722. 20090922. DOI: 10.1038/nrcardio.2009.163 19770848

[6] Korkeila J Vahtera J Korkeila K , et al. Childhood adversities as predictors of incident coronary heart disease and cerebrovascular disease. Heart 2010;96:298–303. DOI: 10.1136/hrt.2009.188250 20194205

[7] Danese A Moffitt TE Harrington H , et al. Adverse childhood experiences and adult risk factors for age-related disease: depression, inflammation, and clustering of metabolic risk markers. Arch Pediatr Adolesc Med 2009;163:1135–1143. DOI: 10.1001/archpediatrics.2009.214 19996051 PMC3560401

[8] Kivimäki M Lawlor DA Smith GD , et al. Early socioeconomic position and blood pressure in childhood and adulthood: the Cardiovascular Risk in Young Finns Study. Hypertension 2006;47:39–44. 20051205. DOI: 10.1161/01.HYP.0000196682.43723.8a 16330678

[9] O’Hare C Kuh D Hardy R. Association of early-life factors with life-course trajectories of resting heart rate: more than 6 decades of follow-up. JAMA Pediatr 2018;172:e175525. 20180402. DOI: 10.1001/jamapediatrics.2017.5525 PMC5875352 29435577

[10] Lehman BJ Taylor SE Kiefe CI , et al. Relationship of early life stress and psychological functioning to blood pressure in the CARDIA study. Health Psychol 2009;28:338–346. DOI: 10.1037/a0013785 19450040 PMC2844101

[11] Izzard AS Heagerty AM. Hypertension and the vasculature: arterioles and the myogenic response. J Hypertens 1995;13:1–4. 7759839

[12] Kähönen E Lyytikäinen LP Aatola H , et al. Systemic vascular resistance predicts the development of hypertension: the Cardiovascular Risk in Young Finns Study. Blood Press 2020;29:362–369. 20200629. DOI: 10.1080/08037051.2020.1783992 32597238

[13] Raitakari OT Juonala M Rönnemaa T , et al. Cohort profile: the Cardiovascular Risk in Young Finns Study. Int J Epidemiol 2008;37:1220–1226. 2008/02/12. DOI: 10.1093/ije/dym225 18263651

[14] Makkonen TR Rönkä T Timonen S , et al. Operation family study. Helsinki: Mannerheim League of Child Welfare, 1981.

[15] Saarinen A Lyytikäinen LP Hietala J , et al. Magical thinking in individuals with high polygenic risk for schizophrenia but no non-affective psychoses – a general population study. Mol Psychiatry 2022 ; 27:3286–3293. DOI: 10.1038/s41380-022-01581-z 10.1038/s41380-022-01581-z PMC9708578 35505089

[16] Keltikangas-Järvinen L Pulkki-Råback L Elovainio M , et al. DRD2 C32806T modifies the effect of child-rearing environment on adulthood novelty seeking. Am J Med Genet B Neuropsychiatr Genet 2009;150b:389–394. 2008/07/11. DOI: 10.1002/ajmg.b.30830 18615478

[17] Pulkki L Keltikangas-Järvinen L Ravaja N , et al. Child-rearing attitudes and cardiovascular risk among children: moderating influence of parental socioeconomic status. Prev Med 2003;36:55–63. DOI: 10.1006/pmed.2002.1125 12473425

[18] Saarinen A Keltikangas-Järvinen L Dobewall H , et al. Childhood family environment predicting psychotic disorders over a 37-year follow-up -a general population cohort study. Schizophr Res 2023;258:9–17. DOI: 10.1016/j.schres.2023.06.008 37392583 10.1016/j.schres.2023.06.008

[19] Saarinen A Marttila S Mishra PP , et al. Polygenic risk for schizophrenia, social dispositions, and pace of epigenetic aging: results from the Young Finns Study. Aging Cell 2024;23(3):e14052. DOI: 10.1111/acel.14052 10.1111/acel.14052 PMC10928579 38031635

[20] Kööbi T Kaukinen S Ahola T , et al. Non-invasive measurement of cardiac output: whole-body impedance cardiography in simultaneous comparison with thermodilution and direct oxygen Fick methods. Intensive Care Med 1997;23:1132–1137. DOI: 10.1007/s001340050469 9434918

[21] Kööbi T Kaukinen S Turjanmaa VM , et al. Whole-body impedance cardiography in the measurement of cardiac output. Crit Care Med 1997;25:779–785. DOI: 10.1097/00003246-199705000-00012 9187596

[22] Koskela JK Tahvanainen A Haring A , et al. Association of resting heart rate with cardiovascular function: a cross-sectional study in 522 Finnish subjects. BMC Cardiovasc Disord 2013;13:102. 20131115. DOI: 10.1186/1471-2261-13-102 24237764 PMC3832902

[23] Raiko JR Viikari JS Ilmanen A , et al. Follow-ups of the Cardiovascular Risk in Young Finns Study in 2001 and 2007: levels and 6-year changes in risk factors. J Intern Med 2010;267:370–384. 20090625. DOI: 10.1111/j.1365-2796.2009.02148.x 19754855

[24] Juonala M Viikari JS Kähönen M , et al. Alcohol consumption is directly associated with carotid intima-media thickness in Finnish young adults: the Cardiovascular Risk in Young Finns Study. Atherosclerosis 2009;204:e93–e98. 20081130. DOI: 10.1016/j.atherosclerosis.2008.11.021 19124122

[25] Gustafsson PE Janlert U Theorell T , et al. Social and material adversity from adolescence to adulthood and allostatic load in middle-aged women and men: results from the Northern Swedish Cohort. Ann Behav Med 2012;43:117–128. DOI: 10.1007/s12160-011-9309-6 22031214 PMC3274686

[26] Woodward M Webster R Murakami Y , et al. The association between resting heart rate, cardiovascular disease and mortality: evidence from 112,680 men and women in 12 cohorts. Eur J Prev Cardiol 2014;21:719–726. 20120620. DOI: 10.1177/2047487312452501 22718796

[27] Jouven X Zureik M Desnos M , et al. Resting heart rate as a predictive risk factor for sudden death in middle-aged men. Cardiovasc Res 2001;50:373–378. DOI: 10.1016/s0008-6363(01)00230-9 11334841

[28] Laitinen TT Puolakka E Ruohonen S , et al. Association of socioeconomic status in childhood with left ventricular structure and diastolic function in adulthood: the Cardiovascular Risk in Young Finns Study. JAMA Pediatr 2017;171:781–787. DOI: 10.1001/jamapediatrics.2017.1085 28655058 PMC5710638

[29] Pulkki-Råback L Elovainio M Hakulinen C , et al. Cumulative effect of psychosocial factors in youth on ideal cardiovascular health in adulthood: the Cardiovascular Risk in Young Finns Study. Circulation 2015;131:245–253. 2015/01/15. DOI: 10.1161/circulationaha.113.007104 25583139

[30] Hakulinen C Pulkki-Råback L Elovainio M , et al. Childhood psychosocial cumulative risks and carotid intima-media thickness in adulthood: the Cardiovascular Risk in Young Finns Study. Psychosom Med 2016;78:171–181. DOI: 10.1097/psy.0000000000000246 26809108 PMC4739501

[31] Wright LB Treiber FA Davis H , et al. Relationship between family environment and children’s hemodynamic responses to stress: a longitudinal evaluation. Behav Med 1993;19:115–121. DOI: 10.1080/08964289.1993.9935180 8292835

[32] Dempster KS O’Leary DD MacNeil AJ , et al. Linking the hemodynamic consequences of adverse childhood experiences to an altered HPA axis and acute stress response. Brain Behav Immun 2021;93:254–263. 20201221. DOI: 10.1016/j.bbi.2020.12.018 33358983

[33] Svensson J Theorell T. Life events and elevated blood pressure in young men. J Psychosom Res 1983;27:445–455. DOI: 10.1016/0022-3999(83)90033-8 6663516

[34] Theorell T Svensson J Knox S , et al. Young men with high blood pressure report few recent life events. J Psychosom Res 1986;30:243–249. DOI: 10.1016/0022-3999(86)90055-3 3723455

[35] Sherwood A Hughes JW McFetridge J. Ethnic differences in the hemodynamic mechanisms of ambulatory blood pressure regulation. Am J Hypertens 2003;16:270–273. DOI: 10.1016/s0895-7061(02)03269-7 12670742

