# Supplementary material for: Childhood family environment and systemic haemodynamics in adulthood: the Cardiovascular Risk in Young Finns Study
Source: Scand J Public Health. 2024 Aug 17;53(8):827–35. doi: 10.1177/14034948241262185 (PMC12619843; doi:10.1177/14034948241262185)
Supplement: sj-docx-1-sjp-10.1177_14034948241262185 – Supplemental material for Childhood family environment and systemic haemodynamics in adulthood: the Cardiovascular Risk in Young Finns Study [file sj-docx-1-sjp-10.1177_14034948241262185.docx]

**Supplementary Table 1.** Results of attrition analyses.

| **Study variable** | **Test statistic** | ***p*** | **Included vs. dropped-out** |
| --- | --- | --- | --- |
| Age | *t* = -1.55 | 0.121 |  |
| Sex (female) | *χ^2^* = 27.83 | **< 0.001** | 55.8 % *vs*. 47.0 % |
|  |  |  |  |
| Stroke volume index 2007 | *t* = 0.27 | 0.785 |  |
| Systemic vascular resistance index 2007 | *t* = -2.61 | **0.009** | 2685.75 *vs*. 2782.39 |
| Heart rate 2007 | *t* = 0.95 | 0.343 |  |
| Cardiac output index 2007 | *t* = 0.99 | 0.324 |  |
|  |  |  |  |
| Socioeconomic family risk 1980 | *t* = -5.95 | **< 0.001** | -0.07 *vs*. 0.07 |
| Adverse emotional family atmosphere 1980 | *t* = -6.73 | **< 0.001** | -0.05 *vs*. 0.05 |
| Stressful life events 1980 | *t* = -11.05 | **< 0.001** | -0.08 *vs*. 0.07 |
| Parents’ risky health behaviors 1980 | *t* = -0.69 | 0.493 |  |
|  |  |  |  |
| Systolic blood pressure 1980 | *t* = -3.03 | **0.003** | 111.86 *vs*. 113.10 |
| HDL cholesterol 1980 | *t* = -0.42 | 0.676 |  |
| LDL cholesterol 1980 | *t* = -1.06 | 0.288 |  |
| Triglycerides 1980 | *t* = -0.47 | 0.636 |  |
| Insulin 1980 | *t* = 0.39 | 0.696 |  |
|  |  |  |  |
| Systolic blood pressure 2007 | *t* = -3.19 | **0.001** | 120.17 *vs*. 122.37 |
| HDL cholesterol 2007 | *t* = -2.39 | **0.017** | 1.33 *vs*. 1.37 |
| LDL cholesterol 2007 | *t* = -3.01 | **0.003** | 3.06 *vs*. 3.18 |
| Triglycerides 2007 | *t* = -4.05 | **< 0.001** | 1.35 *vs*. 1.53 |
| Insulin 2007 | *t* = 0.30 | 0.763 |  |
|  |  |  |  |
| Alcohol use 2007 | *t* = -1.18 | 0.237 |  |
| Physical activity 2007 | *t* = -0.54 | 0.525 |  |
| BMI 2007 | *t* = -1.32 | 0.185 |  |
| Daily smoking (yes) 2007 | *χ^2^ = 0.73* | 0.391 |  |

**Supplementary Table 2.** Results of regression analyses when predicting hemodynamic outcomes in 2007 by cumulative risk scores of family environment in 1980. Adjusted for adulthood age, sex, HDL and LDL cholesterol, triglycerides, and insulin in 2007.

|  | B | *SE* | *p* |
| --- | --- | --- | --- |
| **Stroke volume index (SI)** |  |  |  |
| Socioeconomic family risk | -0.621 | 0.225 | **0.006** |
| Stressful life events | -0.200 | 0.424 | 0.637 |
| Risky emotional atmosphere | 0.501 | 0.342 | 0.144 |
| Parents’ risky health behavior | 0.124 | 0.390 | 0.751 |
| **Systemic vascular resistance index (SVRI)** |  |  |  |
| Socioeconomic family risk | 36.245 | 20.836 | 0.082 |
| Stressful life events | -20.435 | 39.377 | 0.604 |
| Risky emotional atmosphere | -48.263 | 31.761 | 0.129 |
| Parents’ risky health behavior | 21.425 | 36.219 | 0.554 |
| **Heart rate (HR)** |  |  |  |
| Socioeconomic family risk | 1.086 | 0.374 | **0.004** |
| Stressful life events | 0.496 | 0.706 | 0.483 |
| Risky emotional atmosphere | -0.158 | 0.570 | 0.781 |
| Parents’ risky health behavior | -0.629 | 0.650 | 0.333 |
| **Cardiac output index (CI)** |  |  |  |
| Socioeconomic risk | 0.007 | 0.019 | 0.722 |
| Stressful life events | 0.002 | 0.036 | 0.946 |
| Risky emotional atmosphere | 0.032 | 0.029 | 0.275 |
| Parents’ risky health behavior | -0.023 | 0.033 | 0.488 |
| *n* = 1589 | | | |

**Supplementary Table 3.** Results of regression analyses when predicting hemodynamic outcomes in 2007 by cumulative risk scores of family environment in 1980. Adjusted for adulthood age, sex, HDL and LDL cholesterol, triglycerides, insulin and BMI in 2007.

|  | (*n* = 1589) | | |
| --- | --- | --- | --- |
|  | B | *SE* | *p* |
| **Stroke volume index (SI)** |  |  |  |
| Socioeconomic family risk | -0.46 | 0.22 | **0.040** |
| Stressful life events | -0.04 | 0.42 | 0.925 |
| Risky emotional atmosphere | 0.45 | 0.34 | 0.180 |
| Parents’ risky health behavior | 0.37 | 0.39 | 0.343 |
| **Systemic vascular resistance index (SVRI)** |  |  |  |
| Socioeconomic family risk | 18.34 | 20.75 | 0.377 |
| Stressful life events | -35.95 | 38.90 | 0.356 |
| Risky emotional atmosphere | -40.27 | 31.32 | 0.199 |
| Parents’ risky health behavior | -4.44 | 35.98 | 0.902 |
| **Heart rate (HR)** |  |  |  |
| Socioeconomic family risk | 0.93 | 0.38 | **0.014** |
| Stressful life events | 0.37 | 0.71 | 0.604 |
| Risky emotional atmosphere | -0.17 | 0.57 | 0.769 |
| Parents’ risky health behavior | -0.87 | 0.65 | 0.183 |
| **Cardiac output index (CI)** |  |  |  |
| Socioeconomic risk | 0.11 | 0.02 | 0.582 |
| Stressful life events | 0.01 | 0.04 | 0.842 |
| Risky emotional atmosphere | 0.03 | 0.03 | 0.327 |
| Parents’ risky health behavior | -0.02 | 0.03 | 0.607 |

**Supplementary Table 4.** Childhood Family Environment and Systemic Hemodynamics in Adulthood: The Cardiovascular Risk in Young Finns Study. Results of regression analyses when predicting hemodynamic outcomes in 2007 separately by each cumulative risk score of family environment in 1980.

|  | B | *SE* | *p* |
| --- | --- | --- | --- |
| **Stroke volume index (SI)** |  |  |  |
| Socioeconomic family risk | -0.73 | 0.21 | **< 0.001** |
| Stressful life events | -0.32 | 0.33 | 0.332 |
| Risky emotional atmosphere | 0.56 | 0.31 | 0.072 |
| Parents’ risky health behavior | 0.31 | 0.37 | 0.403 |
| **Systemic vascular resistance index (SVRI)** |  |  |  |
| Socioeconomic family risk | 43.88 | 19.43 | **0.024** |
| Stressful life events | -6.89 | 30.50 | 0.821 |
| Risky emotional atmosphere | -41.50 | 28.53 | 0.146 |
| Parents’ risky health behavior | 3.12 | 34.29 | 0.927 |
| **Heart rate (HR)** |  |  |  |
| Socioeconomic family risk | 1.18 | 0.35 | **0.001** |
| Stressful life events | 0.48 | 0.56 | 0.386 |
| Risky emotional atmosphere | -0.19 | 0.52 | 0.711 |
| Parents’ risky health behavior | -0.63 | 0.62 | 0.313 |
| **Cardiac output index (CI)** |  |  |  |
| Socioeconomic risk | 0.00 | 0.02 | 0.877 |
| Stressful life events | 0.00 | 0.03 | 0.882 |
| Risky emotional atmosphere | 0.03 | 0.03 | 0.189 |
| Parents’ risky health behavior | -0.01 | 0.03 | 0.771 |
| Adjusted for age and sex.  Note: Each cumulative risk score is included as a predictor separately. | | | |

**Supplementary Table 5.** Childhood Family Environment and Systemic Hemodynamics in Adulthood: The Cardiovascular Risk in Young Finns Study. Full-adjusted results of regression analyses when predicting hemodynamic outcomes in 2007 by cumulative risk score of family environment in 1980.

|  | B | *SE* | *p* |
| --- | --- | --- | --- |
| **Stroke volume index (SI)** |  |  |  |
| Socioeconomic family risk | -0.49 | 0.23 | **0.037** |
| Stressful life events | -0.03 | 0.44 | 0.943 |
| Risky emotional atmosphere | 0.40 | 0.35 | 0.255 |
| Parents’ risky health behavior | 0.08 | 0.41 | 0.842 |
| **Systemic vascular resistance index (SVRI)** |  |  |  |
| Socioeconomic family risk | 25.14 | 21.74 | 0.248 |
| Stressful life events | -34.25 | 40.81 | 0.401 |
| Risky emotional atmosphere | -34.21 | 32.87 | 0.298 |
| Parents’ risky health behavior | 0.567 | 38.04 | 0.882 |
| **Heart rate (HR)** |  |  |  |
| Socioeconomic family risk | 0.81 | 0.39 | **0.036** |
| Stressful life events | 0.37 | 0.73 | 0.613 |
| Risky emotional atmosphere | -0.17 | 0.59 | 0.774 |
| Parents’ risky health behavior | -0.65 | 0.68 | 0.335 |
| **Cardiac output index (CI)** |  |  |  |
| Socioeconomic risk | 0.00 | 0.02 | 0.904 |
| Stressful life events | 0.01 | 0.04 | 0.862 |
| Risky emotional atmosphere | 0.02 | 0.03 | 0.412 |
| Parents’ risky health behavior | -0.03 | 0.04 | 0.391 |
| Adjusted for age, sex, childhood cardiovascular covariates (systolic blood pressure, HDL and LDL cholesterol, triglycerides, insulin, and BMI), adulthood health behaviors (alcohol consumption, daily smoking status, physical activity), and adulthood cardiovascular covariates (systolic blood pressure, HDL and LDL cholesterol, triglycerides, insulin, and BMI). n = 1472  Note: All cumulative risk scores were included as predictors simultaneously. | | | |
